# Vascular Endothelial Over-Expression of Human Soluble Epoxide Hydrolase (Tie2-sEH Tr) Attenuates Coronary Reactive Hyperemia in Mice: Role of Oxylipins and ω-Hydroxylases

**DOI:** 10.1371/journal.pone.0169584

**Published:** 2017-01-05

**Authors:** Ahmad Hanif, Matthew L. Edin, Darryl C. Zeldin, Christophe Morisseau, John R. Falck, Mohammed A. Nayeem

**Affiliations:** 1 Basic Pharmaceutical Sciences, School of Pharmacy, Center for Basic and Translational Stroke Research. West Virginia University, Morgantown, West Virginia, United States of America; 2 Division of Intramural Research, NIEHS/NIH, Research Triangle Park, North Carolina, United States of America; 3 University of California at Davis, One Shields Avenue, Davis, California, United States of America; 4 Biochemistry, University of Texas Southwestern Medical Center, Dallas, Texas, United States of America; Universita degli Studi di Catania, ITALY

## Abstract

Cytochromes P450 metabolize arachidonic acid (AA) into two vasoactive oxylipins with opposing biologic effects: epoxyeicosatrienoic acids (EETs) and omega-(ω)-terminal hydroxyeicosatetraenoic acids (HETEs). EETs have numerous beneficial physiological effects, including vasodilation and protection against ischemia/reperfusion injury, whereas ω-terminal HETEs induce vasoconstriction and vascular dysfunction. We evaluated the effect of these oxylipins on post-ischemic vasodilation known as coronary reactive hyperemia (CRH). CRH prevents the potential harm associated with transient ischemia. The beneficial effects of EETs are reduced after their hydrolysis to dihydroxyeicosatrienoic acids (DHETs) by soluble epoxide hydrolase (sEH). ω-terminal HETEs are formed by ω-hydroxylase family members. The relationship among endothelial over-expression of sEH (Tie2-sEH Tr), the changes in oxylipins it may produce, the pharmacologic inhibition of ω-hydroxylases, activation of PPARγ, and CRH response to a brief ischemia is not known. We hypothesized that CRH is attenuated in isolated mouse hearts with endothelial sEH over-expression through modulation of oxylipin profiles, whereas both inhibition of ω-hydroxylases and activation of PPARγ enhance CRH. Compared to WT mice, Tie2-sEH Tr mice had decreased CRH, including repayment volume, repayment duration, and repayment/debt ratio (*P* < 0.05), whereas inhibition of ω-hydroxylases increased these same CRH parameters in Tie2-sEH Tr mice. Inhibition of sEH with *t*-AUCB reversed the decreased CRH in Tie2-sEH Tr mice. Endothelial over-expression of sEH significantly changed oxylipin profiles, including decreases in DHETs, mid-chain HETEs, and prostaglandins (*P* < 0.05). Treatment with rosiglitazone, PPARγ-agonist, enhanced CRH (*P* < 0.05) in both Tie2-sEH Tr and wild type (WT) mice. These data demonstrate that endothelial over-expression of sEH (through changing the oxylipin profiles) attenuates CRH, whereas inhibition of ω-hydroxylases and activation of PPARγ enhance it.

## Introduction

Cytochromes P450 (CYPs) metabolize arachidonic acid (AA) through two pathways to produce vasoactive oxylipins: CYP epoxygenases catalyze the epoxidation of AA to form epoxyeicosatrienoic acids (EETs) while CYP ω-hydroxylases hydroxylate AA to form ω-terminal HETEs. EETs are produced in endothelial cells, and have numerous biological functions, including hyperpolarization and relaxation of vascular smooth muscle cells by activating large conductance Ca^2+^-activated K^+^ channels (BK_Ca_) [1, 2]. EETs are short-lived [3], due to rapid hydration by soluble epoxide hydrolase (sEH) to form dihydroxyeicosatrienoic acids (DHETs). DHETs have been reported to have less, more, or equipotent vasodilatory effects to EETs [4, 5]. While sEH is the main pathway of EET metabolism [6], some EETs, such as 5,6- and 8,9-EET, are substrates for the cyclooxygenase (COX) pathway [7]. Different approaches of targeting sEH to study the effects of EETs have been utilized, including genetic deletion [8–11], endothelial expression [12, 13], and pharmacologic inhibition of sEH [11, 14] or CYP epoxygenases [15]. Increased EET generation in mouse cardiomyocytes was shown to exert cardioprotective effects against ischemia/reperfusion injury [8]. CYP4A is one of the ω-hyroxylases involved in metabolizing AA to ω-terminal HETEs, including the potent vasoconstrictor 20-HETE (hydroxyeicosatetraenoic acid) [16]. In addition to regulating EET levels, sEH expression also impacts CYP ω-hydroxylase pathway; as reported by Yadav et al., CYP4A level was increased in the mesenteric arteries of mice with endothelial expression of sEH (Tie2-sEH Tr mice) [12]. sEH activity also affects the levels of other arachidonic- and linoleic- acids derived oxylipins, such as mid-chain hydroxyeicosatetraenoic acids (HETEs), prostaglandins (PGs), epoxyoctadecaenoic acids (EpOMEs), and hydroxyoctadecadienoic acids (HODEs) [11, 17–20]. sEH inhibition was associated with an increased EET/DHET ratio and enhanced coronary reactive hyperemia [11], whereas endothelial over-expression of sEH decreased EET/DHET ratio and impaired endothelium-dependent vasodilation in the cerebral circulation in mice [13]. Genetic variants, such as the human K55R variant allele, have increased sEH activity and are associated with significantly higher risk of coronary heart disease in Caucasians [21]. EpOMEs, at physiological levels, are protective against hypoxia injury [22, 23]. Pretreatment with 12,13-EpOME protected primary cultures of rabbit renal proximal tubular cells against hypoxia/reoxygenation injury, whereas its metabolite 12,13-DiHOME failed to produce the same protective effect [24]. DiHOMEs may have deleterious effects in the heart: at high concentrations, DiHOMEs are cytotoxic to cells in tissue culture [25]; at more physiological concentrations, DiHOMEs can be vasoconstrictive and cardiodepressive [9].

The heart responds to ischemia by temporarily increasing coronary blood flow [26], known as reactive hyperemia (RH) or coronary RH (CRH). The increased blood flow associated with CRH is protective, as it prevents injury or damage due to ischemia by increasing blood, nutrients and oxygen supply to the deprived heart muscle [11]. Compromised CRH is observed in several pathologic conditions affecting the coronary circulation, such as cardiac hypertrophy [27], metabolic syndrome [28], unstable angina, myocardial infarction, and congestive heart failure [29]. In addition to adenosine [27, 30, 31], nitric oxide (NO) [27], K_ATP_ channels [27] and hydrogen peroxide (H_2_O_2_) [27], oxylipins, such as EETs, DHETs, EpOMEs, DiHOMEs, mid-chain HETEs, prostanoids, and HODEs, may mediate CRH [11].

The vasoactive effects of oxylipins in CRH are not well studied. The actions of EETs may be, at least in part, mediated by peroxisome proliferator-activated receptor-gamma (PPARγ) [11, 32–35]. For example, EET-induced aortic relaxation in mice was mediated by PPARγ [10, 33]. The effects of sEH–over-expression, the associated changes in oxylipin profiles, and the pharmacologic inhibition of ω-hydroxylases on CRH in response to short ischemia have not been investigated. We hypothesized that sEH over-expression attenuates CRH through modulation in oxylipin profiles, whereas inhibition of ω-hydroxylases and activation of PPARγ enhance CRH in isolated mouse hearts.

## Materials and Methods

### Animals

The generation of transgenic mice expressing Tie2-driven human sEH in endothelial cells on a C57BL/6 genetic background (Tie2-sEH Tr) was described by Edin et al. [9]. Tie2-sEH Tr and wild type (WT) mice were of the C57BL/6 genetic background, and were generously provided by Dr. Darryl Zeldin, National Institute of Environmental Health Sciences/National Institutes of Health (NIH). All animal care and experimentation protocols were approved and carried out in accordance with the West Virginia University Institutional Animal Care and Use Committee and were in accordance with the principles and guidelines of the NIH’s *Guide for the Care and Use of Laboratory Animals*. Both male and female mice (14–16 wks old) in equal ratio were used in our study. Mice were maintained in cages with a 12:12 h light-dark cycle and free access to standard chow (Cat #2018, Envigo, Indianapolis, IN) and water. Diet 2018 contains 6.2% fat by weight, including 0.7% palmitic, 0.2% stearic, 1.2% oleic %, 3.1% linoleic, and 0.3% linolenic Acids.

### Langendorff-Perfused Heart Preparation

We used the constant pressure mode of the Langendorff isolated heart perfusion as previously described [11]. Tie2-sEH Tr and wild-type mice (14–16 wks.) were euthanized with sodium pentobarbital (100 mg/kg body weight intra-peritoneally). Hearts were excised and immediately placed into heparinized (5 U/mL) ice-cold Krebs-Henseleit buffer containing (in mM) 119.0 NaCl, 11.0 glucose, 22.0 NaHCO_3_, 4.7 KCl, 1.2 KH_2_PO_4_, 1.2 MgSO_4_, 2.5 CaCl_2_, 2.0 pyruvate, and 0.5 EDTA. After removal of the lungs and tissue surrounding the heart, the aorta was rapidly cannulated with a 20-gauge, blunt-ended needle and continuously perfused with 37°C buffer continuously bubbled with [95% O_2_]–[5% CO_2_] at a constant perfusion pressure of 80 mmHg. The left atrium was excised, and a water-filled balloon made of plastic wrap was inserted into the left ventricle through the mitral valve. The balloon was connected to a pressure transducer for continuous measurement of left ventricular developed pressure (LVDP) and heart rate (HR). The heart was then immersed in a water-jacketed perfusate bath (37° C) and left to beat spontaneously. Left ventricular diastolic pressure was adjusted to 2–5 mmHg. A flow transducer was installed above the cannulated aorta for continuous measurement of CF with an ultrasonic flow probe (Transonic Systems, Ithaca, NY). A Power–Lab Chart data acquisition system (AD Instruments, Colorado Springs, CO) was used for data acquisition. Heart function was allowed to stabilize for 30–40 min before initiation of CRH. Only hearts whose CF increased by more than two fold after a 15-second total occlusion were included in the analysis. This was to include only properly functioning hearts that were not damaged during cannulation and baseline perfusion. Hearts with persistent arrhythmias or LVDP <80 mmHg were excluded.

### Coronary Reactive Hyperemic Response

After stabilization for 30–40 minutes, baseline CF, HR, and LVDP were recorded. Hearts were subjected to 15 seconds of total occlusion by closing the valve directly above the cannulated heart to bring forth CRH. After CF returned to pre-CRH baseline levels, post-CRH baseline CF, CF tracing, peak hyperemic flow (PHF), HR, LVDP, repayment volume (RV), and repayment duration (RD) recordings were analyzed for each isolated heart. Investigational drugs were infused into the aortic perfusion line using a microinjection pump (Harvard Apparatus, Holliston, MA) for 15 minutes, after which another CRH was induced and the same parameters analyzed again. Drugs were infused at a rate equivalent to 1% of CF. The final concentrations, after standardization of dose (0.01, 0.1, 1, & 10 μM) response for the various drugs used in this study were 10 μM for rosiglitazone (PPARγ-agonist), *t*-AUCB (*trans*-4-[4-(3-adamantan-1-yl-ureido)-cyclohexyloxy]-benzoic acid (a selective sEH-inhibitor, University of California, Davis) [36], and 1 μM DDMS (dibromo-dodecenyl-methylsulfimide, CYP4A-blocker). These concentrations are equal or lower than used in previous studies: rosiglitazone, 10 μM; [11, 37], *t*-AUCB, 10 μM; [11, 35], DDMS, 1 μM [38].

### Effect of *t*-AUCB on CRH Response

Isolated Tie2-sEH Tr and WT mouse hearts were subjected to 15 sec of total occlusion. Recordings of the first CRH (baseline CF, CF tracing, LVDP, HR, RV, PHF, and RD) were analyzed for each heart and averaged. *t*-AUCB was infused at a final concentration of 10 μM and 1% of CF rate for 15 min, after which another CRH was induced and the same parameters recorded again and analyzed.

### LC–MS/MS Oxylipin Analysis

Levels of oxylipins (5,6-, 8,9-, 11,12- and 14,15-EET, 5,6-, 8,9-, 11,12- and 14,15-DHET, 5-, 8-, 9-, 11-, 12- and 15-HETE, 9,10- and 12,13-EpOME, 9,10- and 12,13-DiHOME, 9- and 13-HODE, 6-keto prostaglandin-F_1α_ [6K-PG-F_1α_], PG-F_2α_, thromboxane B_2_ [TxB_2_], PGD_2_, and PGE_2_) were quantified in pre- and post-CRH heart perfusates of Tie2-sEH Tr and WT mice through liquid chromatography, tandem mass spectroscopy (LC-MS/MS) as described previously [39]. Heart perfusates were collected for 2.5 min after the first 30 min of stabilization and immediately after reperfusion. Hearts were immersed in 5 mL of warm (37°C) Krebs-Henseleit buffer with 5 μL of 10 μM *t*-AUCB to block further EET breakdown by sEH. Heart perfusates were collected two times before ischemia (baseline) and pooled together as one sample and two times after ischemia and pooled together as another sample for LC-MS/MS analysis. Samples were stored at –80°C until processing. Samples were spiked with 30 ng PGE2-d4, 10,11- DiHN, and 10,11-EpHep (Cayman) as internal standards, mixed with 0.1 vol of 1% acetic acid in 50% methanol, and extracted by serial passage through Oasis HLB C18 3mL columns (Waters, Milford, MA, USA). Columns were washed twice with 0.1% acetic acid in 5% methanol and eluted with methanol into glass tubes containing 6 μL of 30% glycerol in methanol. The methanol was then evaporated under a stream of nitrogen gas, and the dried tubes were frozen and stored at –80°C until analysis. Online liquid chromatography of extracted samples was performed with an Agilent 1200 Series capillary HPLC (Agilent Technologies, Santa Clara, CA, USA). Separations were achieved using a Halo C18 column (2.7 mm, 10062.1 mm; MAC-MOD Analytical, Chadds Ford, PA), which was held at 50°C. Mobile phase A was 85:15:0.1 water: acetonitrile: acetic acid. Mobile phase B was 70:30:0.1 acetonitrile: methanol: acetic acid. Flow rate was 400 μL/min; Gradient elution was used. Mobile phase percentage B and flow rate were varied as follows: 20% B at 0 min, ramp from 0 to 5 min to 40% B, ramp from 5 to 7 min to 55% B, ramp from 7 to 13 min to 64% B. From 13 to 19 min the column was flushed with 100% B at a flow rate of 550 μL/min. Samples were solvated in 50 μl of 30% ethanol. The injection volume was 10 μL. Samples were analyzed in triplicate. Analyses were performed on an MDS Sciex API 3000 equipped with a TurboIonSpray source (Applied Biosystems). Turbo desolvation gas was heated to 425°C at a flow rate of 6 L/min. Negative ion electrospray ionization tandem mass spectrometry with multiple reaction monitoring was used for detection. Analyte quantification was performed using Analyst 1.5.1 software (AB Sciex, Ontario, Canada). Relative response ratios of analytes and respective internal standards were compared to a standard curve of response ratios for each analyte. Lipid standards, which are sensitive to oxidative degradation, were stored in 100% ethanol under argon and used within 1 year of purchase from Cayman Chemical (Detroit, MI).

### Effect of DDMS (ω-hydroxylases-inhibitor) on CRH Response

Isolated Tie2-sEH Tr and WT mice hearts were stabilized for 30–40 min, followed by 15 sec of total occlusion. Recordings of the first CRH (baseline CF, CF tracing, LVDP, HR, RV, PHF and RD) were analyzed for each heart and averaged. DDMS was infused at a final concentration of 1.0 μM for 15 minutes, after which the second CRH was induced. CRHs before and after DDMS infusion were analyzed and compared.

### Effect of Rosiglitazone on CRH Response

After stabilization, WT mouse hearts were subjected to 15 sec of total occlusion. As described above, baseline CRH was induced in each mouse heart. Rosiglitazone (Cayman Chemical) was infused at a final concentration of 10 μM for 15 min, followed by another CRH. CRHs before and after rosiglitazone infusion were analyzed and compared.

### Statistical and Data Analyses

Flow debt (baseline flow rate multiplied by occlusion duration) and RV (the integral of hyperemic area above the baseline flow) were calculated using “the integral relative to baseline” function in the data pad of Lab-Chart 7.0 software. Since absolute coronary flow rates change proportionally with heart mass, the RV and flow debt are presented as ml/g wet heart weight, and baseline and peak flow rate data are presented as (mL.min^–1^.g wet heart weight^–1^). Values are means ± standard error; *n* represents the number of animals. For data analysis, two-tailed unpaired *t*-test was used for unpaired data analysis, and two-way ANOVA was used to compare data between groups. Differences were considered statistically significant when *P* < 0.05.

## Results

### CRH Response

#### Effect of sEH endothelial expression on CRH response

**Fig 1:**
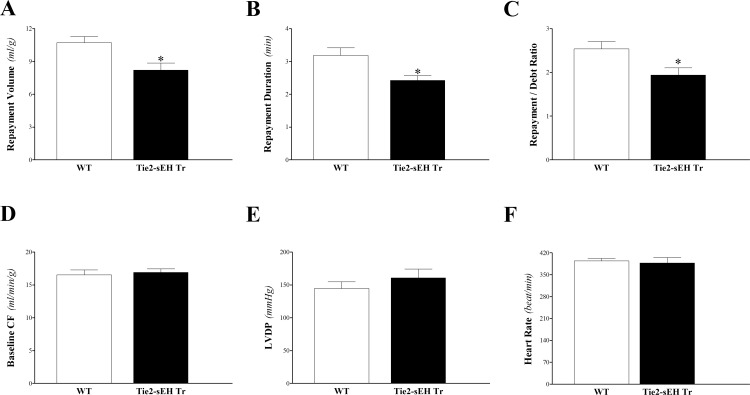
Comparison of coronary reactive hyperemia (CRH) between Tie2-sEH Tr and WT. Repayment volume (A), repayment duration (B), and repayment/debt ratio (C), were increased in Tie2-sEH Tr compared to WT mice (*P* < 0.05). Baseline CF (D), LVPD (E), and HR (F) were not different between the two groups. * *P* < 0.05 versus WT. *n* = 12 per group.

Endothelial expression of sEH attenuated CRH in Tie2-sEH Tr compared to WT mice. Compared to WT mice, Tie2-sEH Tr mice had decreased repayment volume (10.7 ± 0.5 and 8.2 ± 0.6 mL/g, respectively; *P* < 0.05, **Fig 1A**), decreased repayment duration (3.2 ± 0.2 and 2.4 ± 0.2 min, respectively; *P* < 0.05; **Fig 1B**), and decreased repayment/debt ratio (2.5 ± 0.2 and 1.9 ± 0.2, respectively; *P* < 0.05; **Fig 1C**). There was no statistically significant difference in body weight, heart weight, baseline CF, LVPD, and HR between the two groups (*P* > 0.05; **Fig 1D–1F**). **Table 1**lists the statistical mean values and the standard error of the mean (SEM) for these parameters for the two groups of mice. Time-matched control experiments with WT mouse hearts, employing three consecutive inductions of CRH, showed no change in the CRH response and no difference in baseline heart functions, including CF, LVDP, and HR (data not shown).

**Table 1:** Baseline functional data and coronary reactive hyperemia (CRH) parameters for sEH-over-expressed (Tie2-sEH Tr) and wild type (WT) isolated mouse heart.

	WT	Tie2-sEH Tr
**Age, weeks**	16.8 ± 0.3	16.4 ± 0.1
**Body weight, g**	24.7 ± 1.3	23.6 ± 1.8
**Heart weight, mg**	103 ± 4.9	108 ± 5.6
**Repayment volume, mL.g**^**-1**^	10.7 ± 0.5	8.2 ± 0.6 *
**Repayment duration, min**	3.2 ± 0.2	2.4 ± 0.2 *
**Baseline CF, ml.min**^**-1**^**.g**^**-1**^	16.5 ± 0.8	16.9 ± 0.5
**Debt volume, mL.g**^**-1**^	4.3 ± 0.2	4.3 ± 0.1
**Repayment / debt ratio**	2.5 ± 0.2	1.9 ± 0.2 *
**LVDP, mmHg**	145 ± 10	161 ± 13
**Heart rate, beat.min**^**-1**^	394 ± 8	387 ± 17

* *P* < 0.05 versus WT. Values are means ± standard error. *n* = 12 per group.

#### Effect of *t*-AUCB on CRH response in Tie2-sEH Tr and WT mice

**Fig 2:**
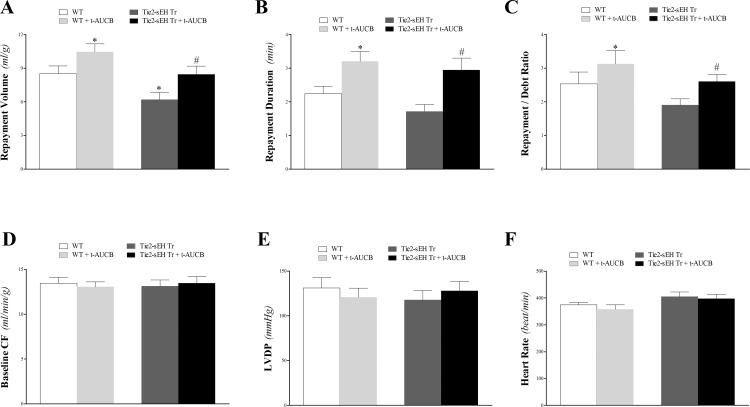
Effect of the sEH-inhibitor (*t*-AUCB, 10 μM) on coronary reactive hyperemia (CRH) in Tie2-sEH Tr and WT mice. The sEH-inhibitor, *t*-AUCB, enhanced CRH in both Tie2-sEH Tr and WT mice. Repayment volume (A), repayment duration (B), and repayment/debt ratio (C) were increased in both strains. Repayment volume was decreased more in Tie2-sEH Tr compared to WT mice. Baseline CF (D), LVPD (E), and HR (F) were not different between the two groups. * *P* < 0.05 versus WT. # *P* < 0.05 versus *t*-AUCB–treated WT. *n* = 8 per group.

The sEH-inhibitor, *t*-AUCB, enhanced CRH in both Tie2-sEH Tr and WT mice. Repayment volume was increased by 23% in WT mice (from 8.5 ± 0.7 to 10.5 ± 0.7 mL/g; *P* < 0.05) compared to 36% in Tie2-sEH Tr mice (from 6.2 ± 0.6 to 8.5 ± 0.7 mL/g; *P* < 0.05, **Fig 2A**). Repayment duration was increased by 43% in WT mice (from 2.2 ± 0.2 to 3.2 ± 0.3 min; *P* < 0.05) compared to 72% in Tie2-sEH Tr mice (from 1.7 ± 0.2 to 3.0 ± 0.4 min; *P* < 0.05, **Fig 2B**). Also, repayment/debt ratio was increased by 23% in WT mice (from 2.5 ± 0.4 to 3.1 ± 0.4; *P* < 0.05) compared to 37% in Tie2-sEH Tr mice (from 1.9 ± 0.2 to 2.6 ± 0.2; *P* < 0.05, **Fig 2C**). There was no significant difference between *t*-AUCB–treated WT and *t*-AUCB–treated Tie2-sEH Tr mice in the above mentioned parameters based on the 2-way ANOVA analyses of interaction between the 2 variables: mouse strain and drug (*t*-AUCB). Baseline CF, LVPD, and HR were not different between and within the two groups (*P* > 0.05; **Fig 2D–2F**).

### Oxylipin Analysis of Heart Perfusate in WT and Tie2-sEH Tr Mice

**Fig 3:**
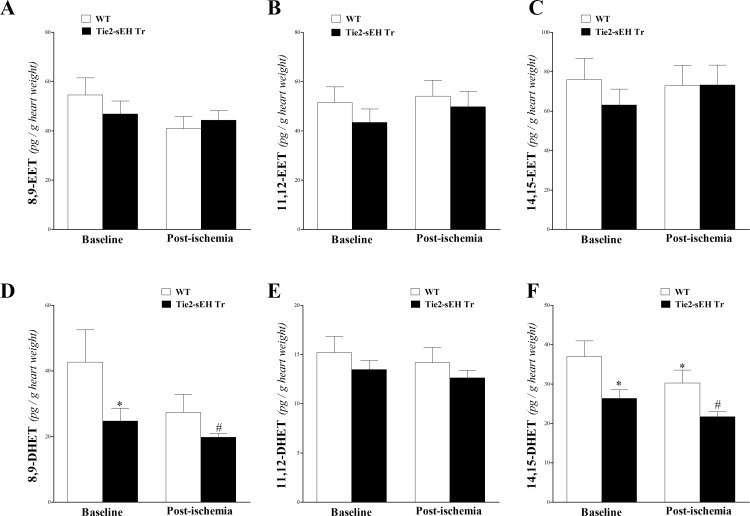
LC–MS/MS analysis for EETs (8, 9–, 11, 12–, and 14, 15–) and DHETs (8, 9–, 11, 12–, and 14, 15–) levels in WT and Tie2-sEH Tr mouse heart perfusates at baseline and post-ischemia. 8,9-EET (A), 11,12-EET (B), and 14,15-EET (C) were not significantly different between WT and Tie2-sEH Tr mice. Also, the levels of EETs were not affected in response to ischemia in either mouse strain (*P* > 0.05). 8,9-DHET (D), and 14,15-DHET (F) were decreased in Tie2-sEH Tr compared to WT mice (*P* < 0.05), whereas 11,12-DHET (E) was not significantly different between the two groups (*P* > 0.05). Also, 14,15-DHET (F) was decreased in response to ischemia in Tie2-sEH Tr (*P* < 0.05). * *P* < 0.05 versus baseline WT. # *P* < 0.05 versus WT post-ischemia. *n* = 12 WT and 14 Tie2-sEH Tr.

Heart perfusate oxylipin levels were determined by LC–MS/MS. Perfusate samples were collected at baseline after stabilization and after ischemia in WT and Tie2-sEH Tr mice. This technique detected 3 out of the 4 EET regioisomers (8,9-, 11,12-, and 14,15-EETs), and their corresponding metabolites (8,9-, 11,12-, and 14,15-DHETs). The measured EETs (8,9-, 11,12-, and 14,15-EETs) were not significantly different between WT and Tie2-sEH Tr mice, (*P* > 0.05; **Fig 3A–3C**). Moreover, these EETs did not significantly change in response to ischemia in either mouse strain (*P* > 0.05; **Fig 3A–3C**). For DHETs, a decreasing trend was noticed in Tie2-sEH Tr compared to WT mice, which was significant in 8,9-, and 14,15-DHETs (*P* < 0.05; **Fig 3D–3F**). Ischemia had a decreasing effect on DHETs, which was significant only in 14,15-DHET in WT mice (*P* < 0.05; **Fig 3F**).

**Fig 4:**
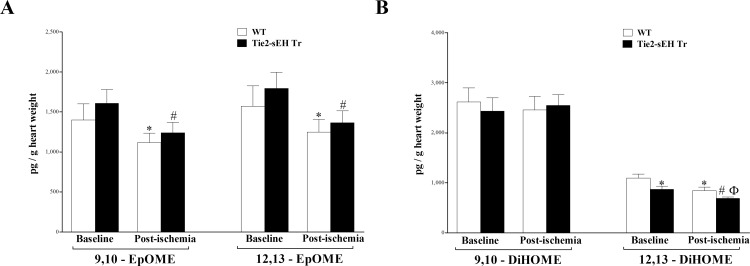
LC–MS/MS analysis of EpOME and DiHOME levels in WT and Tie2-sEH Tr mouse heart perfusates at baseline and post-ischemia. 9,10- and 12,13-EpOMEs (A) were decreased in response to ischemia in both WT and Tie2-sEH Tr mice (*P* < 0.05), but were not different between the two groups (*P* > 0.05). 12, 13-DiHOME (B) level was decreased in Tie2-sEH Tr compared to WT mice (*P* < 0.05), and was decreased in response to ischemia in both mouse groups (*P* < 0.05). 9,10-DiHOME level (B) did not change due to ischemia or to sEH endothelial expression (*P* > 0.05). * *P* < 0.05 versus baseline WT. # *P* < 0.05 versus WT post-ischemia. *n* = 12 WT and 14 Tie2-sEH Tr.

Linoleic acid (LA) epoxides (9,10- and 12,13-EpOMEs) were similar between WT and Tie2-sEH Tr mice, (*P* > 0.05; **Fig 4A**). However, in both strains, these epoxides decreased in response to ischemia (*P* < 0.05; **Fig 4A**). The corresponding 12, 13-DiHOME level was lower in Tie2-sEH Tr compared to WT mice (*P* < 0.05; **Fig 4B**), and was decreased in response to ischemia in both mouse groups (*P* < 0.05; **Fig 4B**). The other DiHOME (9,10-DiHOME) did not change due to ischemia or to sEH endothelial expression (*P* > 0.05; **Fig 4B**).

**Fig 5:**
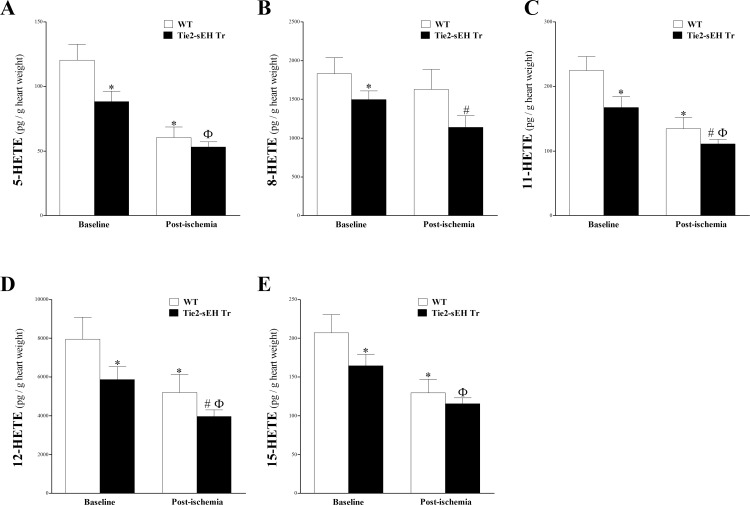
LC–MS/MS analysis of 5-, 8-, 11-, 12- and 15-HETE levels in WT and Tie2-sEH Tr mouse heart perfusates at baseline and post-ischemia. In Tie2-sEH Tr mice, the levels of 5-HETE (A), 8-HETE (B), 11-HETE (C), 12-HETE (D), 15-HETE (E), were decreased compared to WT mice at baseline and post-ischemia (*P* < 0.05). In both WT and Tie2-sEH Tr mice, post-ischemic levels of 5-, 11-, 12-, and 15-HETEs were decreased compared to baseline levels (*P* < 0.05). * *P* < 0.05 versus baseline WT. # *P* < 0.05 versus WT post-ischemia. Ф *P* < 0.05 versus baseline Tie2-sEH Tr. *n* = 12 WT and 14 Tie2-sEH Tr.

Mid-chain HETEs (5-, 8-, 11-, 12-, and 15-HETEs) were detected in WT and Tie2-sEH Tr mouse heart perfusates before and after ischemia. In Tie2-sEH Tr mice, the levels of 5-, 8-, 11-, 12-, and 15-HETE were significantly decreased compared to WT mice at baseline and post-ischemia (*P* < 0.05; **Fig 5A–5E)**. Post-ischemic levels of 5-, and 15-HETEs were not significantly decreased in Tie2-sEH Tr compared to WT mice (*P* > 0.05; **Fig 5A and 5E**). Ischemia also had a decreasing effect on mid-chain HETEs in WT (reaching significant levels for 5-, 11-, 12-, and 15-HETEs) and Tie2-sEH Tr mice (reaching significant levels for 5-, 11-, 12-, and 15-HETEs) (*P* < 0.05; **Fig 5A–5E)**.

**Fig 6:**
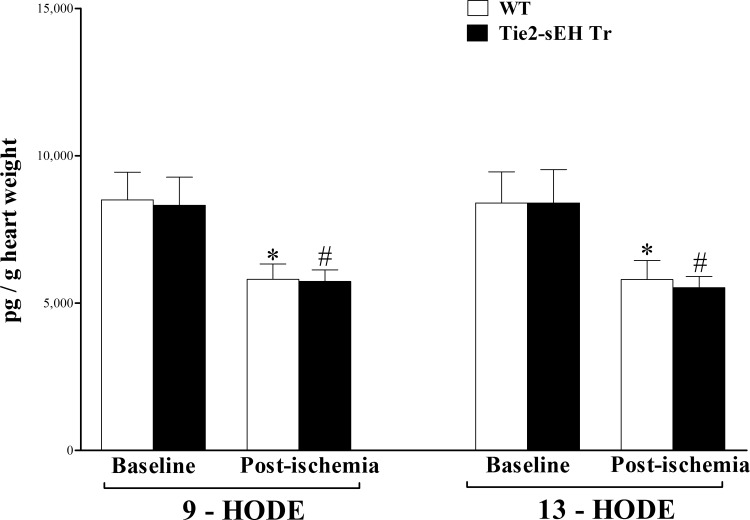
LC–MS/MS analysis of HODEs in WT and Tie2-sEH Tr mouse heart perfusates at baseline and post-ischemia. In both WT and Tie2-sEH Tr, 9- and 13-HODEs decreased in response to ischemia (*P* < 0.05). However, they were not significantly different between the two groups at baseline or post-ischemia (*P* > 0.05). * *P* < 0.05 versus baseline WT. # *P* < 0.05 versus WT post-ischemia. *n* = 12 WT and 14 Tie2-sEH Tr.

Other LA hydroxylated metabolites, 9- and 13-HODEs, were not significantly different between WT and Tie2-sEH Tr mice at baseline or post-ischemia (*P* > 0.05; **Fig 6**). However, in both WT and Tie2-sEH Tr, 9- and 13-HODEs decreased in response to ischemia (*P* < 0.05; **Fig 6**).

**Fig 7:**
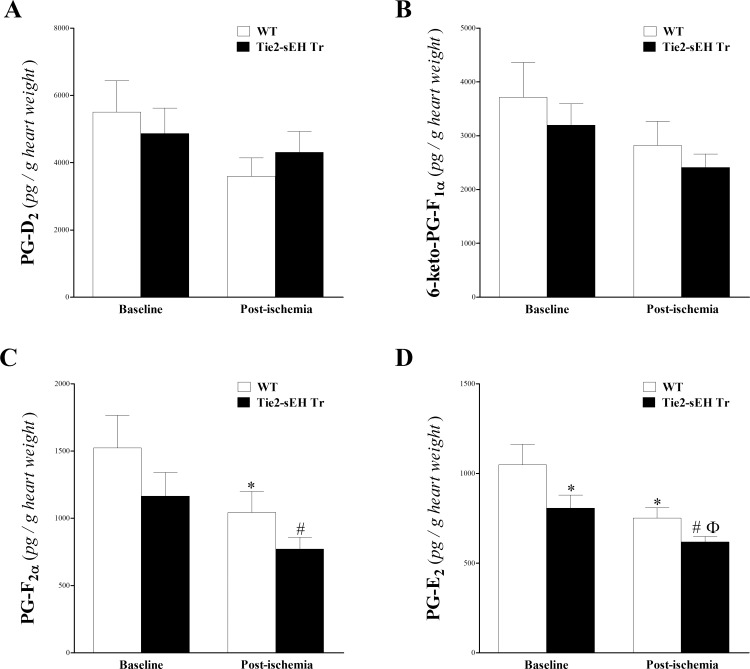
LC–MS/MS analysis of 6-keto-PG-F_1α_, PG-F_2α_, PG-D_2_, and PG-E_2_ in WT and Tie2-sEH Tr mouse heart perfusates at baseline and post-ischemia. The levels of PG-D_2_ (A) and 6-Keto-PG-F_1α_ (B) were not significantly changed due to ischemia or between Tie2-sEH Tr and WT mice (*P* > 0.05). In both Tie2-sEH Tr and WT mice, PG-F_2α_ (C) was decreased post-ischemia (*P* < 0.05). PG-E_2_ (D) was decreased at baseline and post-ischemia in Tie2-sEH Tr compared to WT mice (*P* < 0.05), and it was also decreased in response to ischemia in Tie2-sEH Tr mice (*P* < 0.05). * *P* < 0.05 versus baseline WT. # *P* < 0.05 versus WT post-ischemia. Ф *P* < 0.05 versus baseline Tie2-sEH Tr. *n* = 12 WT and 14 Tie2-sEH Tr.

The levels of 6K-PG-F_1α_, PG-F_2α_, PG-D_2_, and PG-E_2_ were also detected by our LC–MS/MS (**Fig 7**). For these PGs, a decreasing trend in their level was noticed in Tie2-sEH Tr compared to WT mice, which was significant at baseline in PG-E_2_ (*P* < 0.05; **Fig 7D**), and post-ischemia in PG-F_2α_, and PG-E_2_ (*P* < 0.05; **Fig 7C and 7D**). Also, ischemia decreased the level of these PGs in both strains, but was significant in PG-F203B0031, and PG-E_2_ (*P* < 0.05; **Fig 7C and 7D**)

### Effect of DDMS (ω-hydroxylases-inhibitor) on CRH Response

**Fig 8:**
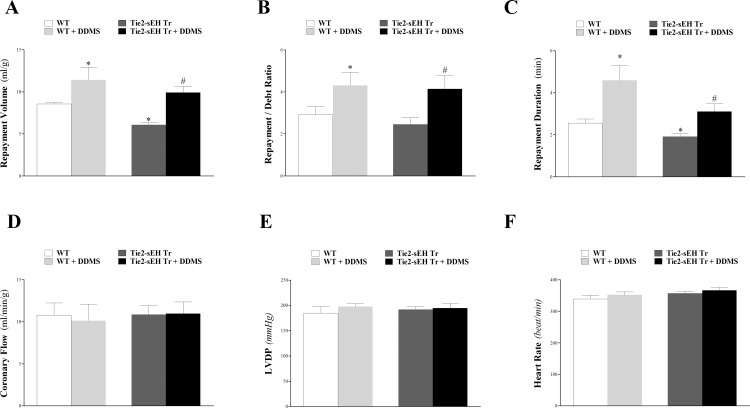
Effect of the CYP4A-blocker (DDMS, 1 μM) on coronary reactive hyperemia (CRH) in Tie2-sEH Tr and WT mice. The CYP4A-blocker, DDMS, enhanced CRH in both Tie2-sEH Tr and WT mice. Repayment volume (A), repayment duration (B), and repayment/debt ratio (C) were increased in both strains. Repayment volume was decreased more in Tie2-sEH Tr compared to WT mice. Baseline CF (D), LVPD (E), and HR (F) were not different between the two groups. * *P* < 0.05 versus WT. # *P* < 0.05 versus DDMS–treated WT. *n* = 8 per group.

DDMS enhanced CRH in both Tie2-sEH Tr and WT mice. Repayment volume was increased by 33% in WT mice (from 8.6 ± 0.2 to 11.4 ± 1.5 mL/g; *P* < 0.05) compared to 63% in Tie2-sEH Tr mice (from 6.1 ± 0.2 to 9.9 ± 0.8 mL/g; *P* < 0.05, **Fig 8A**). Repayment duration was increased by 80% in WT mice (from 2.6 ± 0.2 to 4.6 ± 0.7 min; *P* < 0.05) compared to 62% in Tie2-sEH Tr mice (from 1.9 ± 0.2 to 3.1 ± 0.4 min; *P* < 0.05, **Fig 8B**). Also, repayment/debt ratio was increased by 48% in WT mice (from 2.9 ± 0.4 to 4.3 ± 0.6; *P* < 0.05) compared to 69% in Tie2-sEH Tr mice (from 2.5 ± 0.3 to 4.1 ± 0.7; *P* < 0.05, **Fig 8C**). There was no significant difference between DDMS–treated WT and DDMS–treated Tie2-sEH Tr mice in the above mentioned parameters based on the 2-way ANOVA analyses of interaction between the 2 variables: mouse strain and drug (DDMS). Baseline CF, LVPD, and HR were not different between and within the two groups (*P* > 0.05; **Fig 8D–8F**).

### Effect of rosiglitazone (PPARγ agonist) on CRH Response

**Fig 9:**
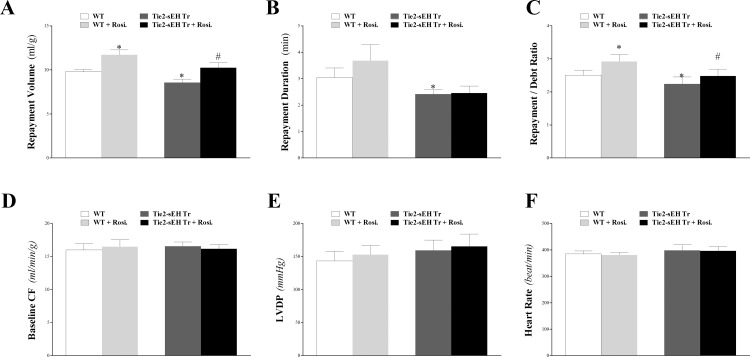
Effect of the PPARγ-agonist (rosiglitazone, 10 μM) on coronary reactive hyperemia (CRH) in Tie2-sEH Tr and WT mice. The PPARγ-agonist, rosiglitazone, enhanced CRH in both Tie2-sEH Tr and WT mice. Repayment volume (A), and repayment/debt ratio (C) were increased in both strains. Repayment duration (B) was increased in WT mice, but not in Tie2-sEH Tr mice. Repayment volume was decreased more in Tie2-sEH Tr compared to WT mice. Baseline CF (D), LVPD (E), and HR (F) were not different between the two groups. * *P* < 0.05 versus WT. # *P* < 0.05 versus rosiglitazone–treated WT. *n* = 8 per group.

Rosiglitazone enhanced CRH in both Tie2-sEH Tr and WT mice. Repayment volume was increased by 19% in WT mice (from 9.8 ± 0.3 to 11.7 ± 0.6 mL/g; *P* < 0.05) compared to 20% in Tie2-sEH Tr mice (from 8.6 ± 0.4 to 10.3 ± 0.6 mL/g; *P* < 0.05, **Fig 9A**). Repayment duration was increased by 21% in WT mice (from 3.1 ± 0.4 to 3.7 ± 0.6 min; *P* < 0.05), but not in Tie2-sEH Tr mice (*P* > 0.05, **Fig 9B**). Repayment/debt ratio, baseline CF, LVPD, and HR were not different between and within the two groups (*P* > 0.05; **Fig 9C–9F**).

## Discussion

Endothelial-specific over-expression of human sEH (Tie2-sEH Tr) in C57BL/6 mice decreased CRH in an isolated heart model in response to brief ischemia. Endothelial over-expression of sEH was also associated with changes in arachidonic and linoleic acids-derived oxylipin profiles. The relationship among sEH over-expression, subsequent oxylipin changes, and modulation of CRH in isolated mouse hearts is not known. Therefore, we designed this study to investigate the role of sEH over-expression and the associated oxylipin changes in the modulation of CRH using isolated WT and Tie2-sEH Tr mouse hearts. Our data demonstrated that: **1)** Endothelial over-expression of sEH decreased CRH; **2)** Inhibition of sEH by *t*-AUCB reversed the decrease in CRH associated with sEH–over-expression in Tie2-sEH Tr mice; **3)** Endothelial over-expression of sEH was associated with changes in some oxylipin profiles, including decrease in DHETs, EpOMEs, DiHOMEs, and mid-chain HETEs; **4)** Inhibition of ω-hydroxylases (by DDMS) enhanced CRH in WT and Tie2-sEH Tr mice; **5)** The PPARγ agonist rosiglitazone enhanced CRH in WT and Tie2-sEH Tr mice.

Endothelial over-expression of human sEH decreased CRH after brief ischemia in Tie2-sEH Tr mice compared to WT mice. Brief ischemia in the heart is followed by CRH [26], which protects the heart against the potential ischemia damage [11]. The significance of CRH was confirmed by several studies which linked compromised CRH with different cardiovascular pathologies [27, 28]. EETs were suggested, among other oxylipins, to mediate CRH in mice in response to brief ischemia [11]. Hydration by sEH is the main metabolic pathway responsible for converting EETs to their primary metabolites, the DHETs [40]. Endothelial over-expression of human sEH, which was associated with increased conversion of EETs to DHETs, impaired endothelium-dependent vasodilation in the cerebral circulation in mice [13]. Lee et al. reported that Caucasian carriers of the K55R variant, with higher sEH activity *in vivo*, had significantly higher risk of incident coronary heart disease [21]. These reports reiterate the well-established beneficial cardiovascular effects of EETs [10, 25, 40–42], including vasodilation in the kidney preglomerular vasculature [43], intestines [44], and brain [45], and protection against ischemia/reperfusion injury [8]. Although DHETs are generally viewed as less active compared to EETs, some studies have reported equal or even more potent vasodilatory effect for DHETs compared to EETs [4, 5, 46]. For example, 11,12- and 14,15-DHETs were shown to cause vasodilation in isolated human coronary arteries [5] and bovine coronary arteries [4] respectively. The relative potency of these EETs and DHETs was also compared, with 11,12-DHET being equipotent to 11,12-EET [5], and 14,15-DHET being fivefold less potent than 14,15-EET [4]. We have previously shown that targeting sEH, through pharmacologic inhibition [11] or genetic deletion [11], was associated with enhanced CRH. In this study, pharmacologic inhibition of sEH, by *t*-AUCB, not only enhanced CRH as previously shown, it also reversed the decrease in CRH observed in Tie2-sEH Tr mice, and increased it to a level comparable to that in WT mice. This approach of targeting EETs pathway from different angles strongly supports previous [11] as well as current findings of sEH involvement in CRH, where inhibiting or deleting sEH [11] enhances CRH, and over-expressing it attenuates CRH.

Our functional data in this study agree with our previously published data [11] in terms of the effect of sEH on CRH. However, the oxylipin data are not as congruent. In the current study, endothelial-specific over-expression of sEH (Tie2-sEH Tr) was, unexpectedly, associated with decreased levels of DHETs compared to WT mice. DHETs are metabolic products of EETs’ hydrolysis by sEH. Our lab has reported that global deletion of sEH (sEH^–/–^mice) [11] and pharmacologic inhibition by *t*-AUCB [11] resulted in decreased DHETs. In contrast to our findings in this study, over-expressing sEH in Tie2-sEH Tr mice resulted in decreased plasma level of 11,12- and 14,15-DHETs compared to WT [13]. Possible explanations for these different results are the different source of the samples: Zhang et al. used plasma samples, whereas we used heart perfusate samples. Another likely possibility is that increasing sEH only in endothelial cells has merely a small effect on total cardiac EET hydrolysis. Cardiomoyctes, which highly express both CYP epoxygenases and sEH [47] comprise 60% of all heart cells [48]. While endothelial cells (ECs) from Tie2-sEH Tr mice have increased endothelial sEH expression and EET hydrolysis [9, 49] (they represent less than 10% of all heart cells [48]. Thus, the effect of endothelial sEH overexpression on total EET and DHET levels in the heart may be minor in the context of hearts with otherwise WT sEH expression. While not altering global cardiac levels of EETs or DHETs, increased sEH in endothelial cells of Tie2-sEH Tr hearts may increase the hydrolysis of EETs in endothelial cells and reduce their paracrine, vasodilatory effects on smooth muscle. We previously reported that global deletion of sEH (sEH^–/–^mice) increased 14,15-EET in heart perfusate samples [11], whereas in this study no difference was found in EET levels between hearts from WT and Tie2-sEH Tr mice. Additionally, the short half-life of EETs [3], and the limited over-expression of sEH to the endothelium in Tie2-sEH Tr mice may explain the lack of change in EET levels in our study. Therefore, the decreased levels of DHETs in our data, which indicates reduced DHET-mediated vasodilation as reported previously [4, 5], may explain the decreased CRH in Tie2-sEH Tr compared to WT mice.

In early studies, the role of sEH in cardiovascular biology was primarily linked to its role in EET hydrolysis; however, sEH also plays a central role in the metabolism and actions of other arachidonic-, linoleic- and omega-3-derived oxylipins [24]. Accordingly, we have expanded oxylipin analyses to include more oxylipins besides EETs and DHETs. The other oxylipins we examined are EpOMEs, which are hydrolyzed to DiHOMEs by sEH. EpOMEs and DiHOMEs are found in abundance in mouse tissues and plasma since their parental fatty acid, linoleic acid, comprises 50% of all fatty acids in our mouse diets. We expected either increase or no-change in DiHOMEs in our Tie2-sEH Tr mice; however, we found no change in 9,10-DiHOME and a decrease in 12,13-DiHOME in Tie2-sEH Tr versus WT mice. In addition, EpOME levels were not different between the two mouse strains. In contrast, we previously found that global deletion of sEH (sEH^–/–^mice) [11] and pharmacologic inhibition of sEH by *t*-AUCB [11] increased EpOME, decreased DiHOMEs, and increased EpOME/DiHOME ratio. In these studies, the altered EpOME/DiHOME ratio may have contributed to the enhancement of CRH [11]. On the other hand, decreased EpOME/DiHOME ratio in the plasma of Tie2-sEH Tr mice was associated with impaired endothelium-dependent vasodilation in the cerebral circulation [13]. These data suggest that a high EpOME/DiHOME ratio may have a positive role in mediating vasodilation, whether the ratio is increased due to elevated EpOMEs [11] and / or decreased DiHOMEs [11, 13]. Compared to EETs, the functions of EpOMEs and DiHOMEs are less well-defined [17]; however, a few studies suggest that EpOMEs, at physiological levels, protected against hypoxia/reoxygenation injury [22, 23]. Konkel et al. demonstrated that pretreatment with 12,13-EpOME protected primary cultures of rabbit renal proximal tubular cells against hypoxia/reoxygenation injury, whereas its metabolite 12,13-DiHOME failed to produce the same protective effect [24]. In contrast, the hydrated metabolites, DiHOMEs, have shown deleterious effects, including cytotoxic, vasoconstrictive and cardiodepressive properties [9, 25]. In our study, the unchanged level of EpOMEs and the modest decrease in 12,13-DiHOME in Tie2-sEH Tr may suggest that they did not contribute much to the decreased CRH in Tie2-sEH Tr compared to WT mice.

Mid-chain (5-, 8-, 11-, 12- and 15-) HETE levels were decreased in Tie2-sEH Tr compared to WT mice and in response to ischemia. Mid-chain HETEs are produced from AA by allylic oxidation by lipoxygenase [24] and by bis-allylic oxidation by CYP1B1 [50]. They were shown to have vasoconstrictive and pro-inflammatory effects [50, 51]. Also, the increased formation of mid-chain HETEs was involved in cardiovascular dysfunction [52–55]. Our finding that mid-chain HETEs were decreased in both mouse strains post-ischemia is in agreement with our previously published data [11]. However, our finding that CRH was decreased in Tie2-sEH Tr mice is not supported by the decreased levels of the vasoconstrictive mid-chain HETEs in the same mice. This surprising result also contrasts with previous findings from our lab in which sEH inhibition was accompanied by decreased mid-chain HETEs in the heart perfusate of WT mice [11]. However, to the best of our knowledge, the level of mid-chain HETEs in Tie2-sEH Tr mice has not been evaluated in any tissue or fluid, including the heart perfusate. Changes in the level of AA metabolites in response to genetic manipulation of relevant enzymes have been reported [11, 18]. This unexpected finding could be reconciled with our functional finding of decreased CRH in Tie2-sEH Tr mice if we consider another important finding: the level of CYP4A. CYP4A is an ω-hyroxylase involved in metabolizing AA to the potent vasoconstrictor 20-HETE [16]. Yadav et al. has recently reported that CYP4A level was increased in the mesenteric arteries of Tie2-sEH Tr mice [12]. These data suggest that while the decrease in mid-chain HETEs observed in the heart perfusates of Tie2-sEH Tr mice should have enhanced CRH, the reported increased expression of CYP4A in the same mice may have overridden the effect of decreased mid-chain HETEs and caused a net effect of decreased CRH.

In addition to EpOMEs, HODEs are the other oxylipins derived from linoleic acid (LA) through hydroxylation by CYP epoxygenases or lipoxygenases [24]. The detected two HODE isomers, 9-, and 13-HODE, were similar in Tie2-sEH Tr and WT. The physiologic functions of HODEs are not widely investigated [24], though 9-, and 13-HODE appear to have some opposing effects [56]. 13-HODE has been suggested to have an anti-inflammatory role [57–61], while 9-HODE was described as pro-inflammatory [62, 63]. Similar to our findings, Luria et al. reported no change in mouse urinary samples of 9– and 13–HODE when sEH was deleted [17]. However, heart perfusate samples from the same strain (sEH^–/–^mice) had increased level of 13–HODE compared to WT [11]. Since endothelial over-expression of sEH in Tie2-sEH Tr mice did not change the level of HODEs, we do not expect that changes in HODEs played any role in the decreased CRH noticed in Tie2-sEH Tr compared to WT mice.

Endothelial over-expression of sEH in Tie2-sEH Tr mice resulted in significant changes in prostanoid levels, including PG-E_2_, and PG-F_2α_. Although PG-F_1α_, and PG-D_2_, had a decreasing trend in Tie2-sEH Tr mice, the difference was not significant. PGs have generally been described as pro-inflammatory [64], still, recent reports indicate that some, such as PG-D_2_ and PG-E_2_, are anti-inflammatory by up-regulating cAMP and inducing secretion of the anti-inflammatory IL-10 [64, 65]. 6-keto-PG-F_1α_ is the non-active metabolite of prostacyclin (PG-I_2_) [64]. Similar to these findings, published reports by our lab and others have demonstrated that pharmacologic inhibition or genetic deletion of sEH were accompanied by decreased prostanoids [11, 19, 20]. Hellmann et al. suggested that prostaglandins are not involved in post-occlusive reactive hyperemia in the skin [66]. Despite the observed decrease in the level of some PGs in Tie2-sEH Tr mice, the lack of evidence for the involvement of PGs in reactive hyperemia and their variable effects on inflammation suggest that PG changes did not likely contribute to the decrease in CRH in Tie2-sEH Tr mice.

As mentioned earlier, ω-terminal HETEs are generated from AA by CYP ω-hydroxylases, primarily CYP4A and CYP4F subfamilies, with 20-HETE being the primary product [16]. 20-HETE is involved with the renin-angiotensin system to promote hypertension, vasoconstriction, and vascular dysfunction [67, 68]. Weldmann et al. suggested that 20-HETE contributes to postmenopausal hypertension in spontaneously hypertensive rats (SHR) [69]. Blocking 20-HETE synthesis by 1-aminobenzotriazole inhibited renal production of 20-HETE, and reduced mean arterial pressure in old female SHR [69]. The changes in the renal production of 20-HETE was consistent with the expression level of CYP4A protein [70]. In this study, inhibiting ω-hydroxylases by DDMS enhanced CRH in Tie2-sEH Tr and WT mice, however, the percent increase in CRH in Tie2-sEH Tr mice (63%) was higher than that in WT mice (33%), which suggests a possible increased activity of ω-hydroxylases in Tie2-sEH Tr compared to WT mice. This in turn could lead to higher levels of 20-HETE. This possibility is supported by the finding that CYP4A’s expression was increased in the mesenteric arteries of Tie2-sEH Tr mice [12]. The levels of 20-HETE were too low to be detected in heart perfusates from WT or Tie2-sEH Tr mice. Upregulation of CYP4A [12], may contribute to reduced CRH in Tie2-sEH Tr compared to WT mice. As mentioned earlier, the temporary pharmacological inhibition of sEH could not completely restore CRH in Tie2-sEH Tr to that in WT mice. This suggests that other mechanisms or mediators, besides sEH, could be involved in decreasing CRH. One such mechanism is the upregulation of CYP4A reported in Tie2-sEH Tr mice and the possible increase in 20-HETE. Improved techniques for detection of the level of 20-HETE in mice are required to better characterize the role it may play in CRH in WT or Tie2-sEH Tr hearts. Nevertheless, our data suggest that dual inhibition of sEH and omega hydroxylases may have a synergistic effect to enhance CRH.

This study also showed that CRH was increased by rosiglitazone, a PPARγ-agonist. PPAR agonists are well known inducers of both sEH [71] and omega hydroxylases [72]. Due to the short treatment periods, and that such induction would likely reduce CRH, we believe induction of these enzymes by rosiglitazone was minimal. Previously, we suggested that PPARγ may mediate CRH downstream of the CYP epoxygenase-EET pathway [11]. Other studies indicated that PPARγ receptors could mediate EETs’ effects [32–35]. Liu et al. suggested that selective sEH inhibition, which increases the retention of EETs, potentiates the anti-inflammatory effect in endothelial cells by PPARγ activation [32]. Although our results suggest that PPARγ receptors are involved in modulating CRH in both Tie2-sEH Tr and WT mice, they also show that the role these nuclear receptors play in CRH is comparable between the two mouse strains, and that endothelial expression of sEH does not impact their role compared to that in WT mice.

In summary, the findings of this study suggest that endothelial over-expression of sEH decreases CRH possibly through attenuating the CYP epoxygenase pathway and augmenting the CYP ω-hydroxylase pathway, as reflected by the changes in AA–derived oxylipins (decreased DHETs, decreased mid-chain HETEs, and decreased PGs) and LA–derived oxylipins (decreased 12,13-DiHOME, and no change in HODEs). The effects of sEH–over-expression on these pathways might have collectively accounted for the observed decrease in CRH. Changes in CRH are partially mediated by PPARγ activation in the two studied mouse strains, as demonstrated by the enhanced CRH after PPARγ-agonist treatment. Therefore, we conclude that sEH–over-expression attenuates, whereas ω-hydroxylases–inhibition and activation of PPARγ enhance CRH.
